# Machine learning algorithms to estimate 10-Year survival in patients with bone metastases due to prostate cancer: toward a disease-specific survival estimation tool

**DOI:** 10.1186/s12885-022-09491-7

**Published:** 2022-04-30

**Authors:** Ashley B. Anderson, Clare Grazal, Rikard Wedin, Claire Kuo, Yongmei Chen, Bryce R. Christensen, Jennifer Cullen, Jonathan A. Forsberg

**Affiliations:** 1 Division of Orthopaedics, Department of Surgery, Uniformed Services University, Walter Reed National Military Medical Center, 8901 Rockville Pike, Bethesda, MD 20889 USA; 2 The Henry Jackson Foundation for the Advancement of Sciences, 6720A Rockledge Dr, Suite 100, Bethesda, MD 20817 USA; 3 Department of Molecular Medicine and Surgery (MMK), K1, Orthopaedics, Karolinska, Institutet, A2:07 171 76, Stockholm, Sweden; 4 Center for Prostate Disease Research, Department of Surgery, Uniformed Services University, Walter Reed National Military Medical Center, 6720A Rockledge Dr, Suite 300, Bethesda, MD 20817 USA; 5 Department of Internal Medicine, San Antonio Military Medical Center, 3551 Roger Brooke Dr, San Antonio, TX 78219 USA; 6 Department of Population and Quantitative Health Sciences, School of Medicine, Case Western Reserve University, Wolstein Research Building 2520, 2103 Cornell Road, Cleveland, OH 44106 USA; 7 Department of Orthopaedic Surgery, The Johns Hopkins University Hospital, 601 N. Caroline St, Baltimore, MD 21287 USA

**Keywords:** Bone metastasis, Machine learning, Oncology, PATHFx, Prostate cancer, Skeletal-related event, Survival estimates

## Abstract

**Background:**

Prognostic indicators, treatments, and survival estimates vary by cancer type. Therefore, disease-specific models are needed to estimate patient survival. Our primary aim was to develop models to estimate survival duration after treatment for skeletal-related events (SREs) (symptomatic bone metastasis, including impending or actual pathologic fractures) in men with metastatic bone disease due to prostate cancer. Such disease-specific models could be added to the PATHFx clinical-decision support tool, which is available worldwide, free of charge. Our secondary aim was to determine disease-specific factors that should be included in an international cancer registry.

**Methods:**

We analyzed records of 438 men with metastatic prostate cancer who sustained SREs that required treatment with radiotherapy or surgery from 1989–2017. We developed and validated 6 models for 1-, 2-, 3-, 4-, 5-, and 10-year survival after treatment. Model performance was evaluated using calibration analysis, Brier scores, area under the receiver operator characteristic curve (AUC), and decision curve analysis to determine the models’ clinical utility. We characterized the magnitude and direction of model features.

**Results:**

The models exhibited acceptable calibration, accuracy (Brier scores < 0.20), and classification ability (AUCs > 0.73). Decision curve analysis determined that all 6 models were suitable for clinical use. The order of feature importance was distinct for each model. In all models, 3 factors were positively associated with survival duration: younger age at metastasis diagnosis, proximal prostate-specific antigen (PSA) < 10 ng/mL, and slow-rising alkaline phosphatase velocity (APV).

**Conclusions:**

We developed models that estimate survival duration in patients with metastatic bone disease due to prostate cancer. These models require external validation but should meanwhile be included in the PATHFx tool. PSA and APV data should be recorded in an international cancer registry.

## Introduction

In the United States, prostate cancer is the most common diagnosed malignancy and the second leading cause of cancer death in men [1, 2]. The clinical treatment decision-making process is challenging because prostate cancer is a complex disease. Several tumor markers and biomarkers are associated with prognosis. For example, proximal prostate-specific antigen (PSA) (defined as the most recent value measured at least 6 months before developing metastasis) < 10 ng/mL is an independent predictor of metastasis-free survival among men with biochemical recurrence after undergoing radical prostatectomy [3]. In addition, the change in alkaline phosphatase concentration over time, alkaline phosphatase velocity (APV), is a prognostic biomarker associated with overall survival in men with castration-resistant prostate cancer [4, 5]. Metwalli et al. [5] found that higher APV was also an independent predictor of overall survival, as well as for bone metastasis–free survival in patients with castration-resistant prostate cancer, where APV ≥ 50 (upper quartile) is “quick rising”, APV of 0 is “no rising”, and all other APV values are “slow rising.” High APV (uppermost quartile of velocity) is also predictive of distant metastasis–free survival in patients who have undergone radical prostatectomy and experienced biochemical recurrence [4].

The approach to treating men with metastatic bone disease due to prostate cancer requires balanced consideration of clinical benefits, life expectancy, comorbidities, quality of life, and the risk of adverse effects. Clinical practice guidelines [6] published recently by the Musculoskeletal Tumor Society recommend that physicians consider using clinical support tools, such as PATHFx, available worldwide at no cost at www.pathfx.org. The tool is designed to estimate a patient’s survival trajectory by estimating survival after treatment for a skeletal-related event (SRE), which is defined as pathologic fracture; spinal cord compression requiring surgical treatment; or nonsurgical treatment, including radiotherapy, cryotherapy, or radiofrequency ablation. PATHFx currently estimates the likelihood of survival at 1, 3, 6, 12, 18 and 24 months after surgical or nonsurgical intervention or an SRE [7–11]. However, patients with metastatic prostate cancer often live much longer than 24 months after an SRE. For this reason, disease-specific models should be developed to augment—or take the place of—the generic models currently powering the PATHFx decision-making support tool. Its use supports shared decision making by ensuring that treatment strategies align with each patient’s personal survival trajectory and functional goals.

PATHFx has been externally validated in several centers worldwide, continual advances in the treatment of patients with advanced cancer require that the models be updated regularly. For this reason, we updated the six PATHFx models using recent data obtained from patients undergoing contemporary systemic therapy, including targeted agents, and immunotherapy [12]. Validation data for this study were derived from the International Bone Metastasis Registry, which helps ensure that the updated models are applicable to various patient populations worldwide. This commitment to lifecycle management ensures that PATHFx remains applicable as treatment philosophies change and new therapies become available, thereby providing clinicians with the most current, broadly applicable tool to estimate survival in this patient population.

Currently, the PATHFx tool groups cancer diagnoses according to historical data on survival rates. Because prognostic indicators, treatment protocols, and survival estimates vary widely by cancer type, it may be beneficial to develop disease-specific survival models. Such models would make use of prognostic information unique to men with metastatic prostate cancer, such as proximal PSA and APV.

Our primary purpose was to develop models to estimate the duration of survival after treatment for skeletal-related events (SREs) in men with metastatic bone disease due to prostate cancer. Such models could inform the PATHFx clinical decision support tool, which currently groups cancer types according to historical survival data. Our secondary purpose was to determine disease-specific factors that should be included in an international cancer registry.

## Methods

### Guidelines

This retrospective prognostic classification study followed the Transparent Reporting of a Multivariable Prediction Model for Individual Prognosis or Diagnosis guidelines [13] and the Guidelines for Developing and Reporting Machine Learning Predictive Models in Biomedical Research [14].

### Data source and patient selection criteria

The study population comprised 29,000 men enrolled in the institutional review board–approved Center for Prostate Disease Research (CPDR) Multicenter National Database Program [15]. We reviewed the records of men who sustained a SRE due to metastatic prostate cancer and subsequently required treatment with radiotherapy or surgery between 1989 and 2017. There were 1,404 men with prostate cancer that metastasized to bone in the data set. Of these, 438 patients had sufficient information to calculate APV, defined as the slope of the linear regression line of alkaline phosphatase values obtained after the diagnosis of metastatic bone disease, plotted against time in years.

### Outcome

We developed six models designed to estimate the likelihood of survival at 1, 2, 3, 4, 5, and 10 years after treatment of an SRE.

### Demographic, clinical, and pathologic features

Consistent with previous methods of using APV as a prognostic feature, and because of the strong skew and non-normality of the APV distribution, APV was binned into the uppermost quartile (“quick-rising”) of all observed values and then compared with the lower 3 quartiles combined (“slow-rising”) and zero value (“no-rising”) [4, 5]. Proximal PSA, defined as PSA concentration at the time of diagnosis of metastatic bone disease, was missing in 11% of these records. Data for all other features were complete. For each consecutive time point, the number of patients decreased because of censoring. Patient demographic and clinical data extracted for analysis were as follows: self-reported race (black or white/other), presence of comorbidities, age at first known bone metastasis, proximal PSA, APV values, method of local treatment of the primary tumor (radiotherapy or surgery), adjuvant therapy (radiotherapy, chemotherapy, and hormonal therapy) and date of death.

Categorical and continuous features included in the models and the proportions of missing data are listed in Tables 1 and 2. We used Bayes factor (BF) analysis to compare the cohorts. BF analysis considers the strength of evidence supporting or contradicting the study hypothesis. The analysis is categorized by the following: BF ≥ 100 indicates strong supporting evidence for the alternative hypothesis; BF < 100 indicates strong supporting evidence for the null hypothesis; and BF of approximately 0 indicates no probable difference between the 2 groups [16, 17].

**Table 1:** Continuous variables contained within the train and test sets

Variable by Time Point	Median (IQR)			*P* value*	Bayes Factor
	Whole Cohort	Train Set	Test Set		
Proximal PSA					
1-Year	33.4 (200)	38.6 (202)	23.7 (153)	0.37	0.15
2-Year	35.7 (205)	30.5 (147)	51.7 (294)	0.59	0.14
3-Year	36.7 (211)	34.0 (205)	46.2 (238)	0.38	0.15
4-Year	36.8 (217)	36.8 (232)	37.4 (202)	0.43	0.15
5-Year	39.6 (236)	42.8 (232)	31.5 (242)	0.54	0.14
10-Year	42.8 (252)	44.5 (275)	40.7 (131)	0.21	0.17
Age					
1-Year	71.0 (12.7)	71.1 (12.6)	70.1 (12.9)	0.41	0.19
2-Year	71.0 (12.7)	71.2 (12.6)	70.6 (12.4)	0.96	0.13
3-Year	71.0 (12.7)	71.0 (12.9)	71.1 (11.7)	0.95	0.13
4-Year	71.0 (12.8)	71.0 (12.7)	71.6 (13.4)	0.32	0.22
5-Year	71.0 (12.9)	70.8 (12.7)	71.5 (13.1)	0.46	0.18
10-Year	71.0 (13.0)	70.8 (12.6)	72.5 (13.7)	0.21	0.32

*PSA* prostate-specific antigen

^*^P values determined using Pearson’s chi-squared test

**Table 2:** Categorical variables contained within the train and test sets

Variable by Time Point	Whole Cohort		Train Set		Test Set		*P* value^*^	Bayes Factor
**Model Features**								
**Comorbidity**	**Yes**	**No**	**Yes**	**No**	**Yes**	**No**		
1-Year	145	293	118	232	27	61	0.68	0.20
2-Year	144	286	112	232	32	54	0.49	0.26
3-Year	143	283	119	221	24	62	0.26	0.39
4-Year	139	278	115	218	24	60	0.36	0.31
5-Year	137	265	108	213	29	52	0.81	0.20
10-Year	127	246	103	195	24	51	0.78	0.21
**Hormone therapy/chemotherapy**								
1-Year	282	156	222	128	60	28	0.48	0.25
2-Year	276	154	230	114	46	40	0.03	2.55
3-Year	272	154	217	123	55	31	> 0.99	0.19
4-Year	266	151	213	120	53	31	0.98	0.19
5-Year	260	142	208	113	52	29	> 0.99	0.19
10-Year	243	130	197	101	46	29	0.52	0.27
**Treatment-naïve**								
1-Year	88	350	76	274	12	76	0.12	0.63
2-Year	87	343	66	278	21	65	0.35	0.28
3-Year	87	339	69	271	18	68	> 0.99	0.16
4-Year	86	331	66	267	20	64	0.51	0.23
5-Year	82	320	66	255	16	65	0.99	0.16
10-Year	77	296	60	238	17	58	0.75	0.19
**Radiotherapy**								
1-Year	68	370	52	298	16	72	0.55	0.19
2-Year	67	363	48	296	19	67	0.09	0.76
3-Year	67	359	54	286	13	73	0.99	0.14
4-Year	65	352	54	279	11	73	0.59	0.17
5-Year	60	342	47	274	13	68	0.89	0.15
10-Year	53	320	41	257	12	63	0.76	0.17
**APV of 0**								
1-Year	188	250	154	196	34	54	0.43	0.28
2-Year	183	247	142	202	41	45	0.34	0.34
3-Year	180	246	149	191	31	55	0.24	0.44
4-Year	176	241	144	189	32	52	0.47	0.27
5-Year	172	230	138	183	34	47	0.97	0.20
10-Year	156	217	123	175	33	42	0.77	0.22
**Quick-rising APV**								
1-Year	111	327	86	264	25	63	0.55	0.22
2-Year	109	321	90	254	19	67	0.52	0.22
3-Year	109	317	82	258	27	59	0.21	0.44
4-Year	108	309	88	245	20	64	0.73	0.19
5-Year	106	296	87	234	19	62	0.60	0.21
10-Year	103	270	86	212	17	58	0.35	0.32
**Slow-rising APV**								
1-Year	139	299	110	240	29	59	0.88	0.19
2-Year	138	292	112	232	26	60	0.78	0.19
3-Year	137	289	109	231	28	58	> 0.99	0.18
4-Year	133	284	101	232	32	52	0.22	0.46
5-Year	124	278	96	225	28	53	0.50	0.26
10-Year	114	259	89	209	25	50	0.66	0.23
**Black**								
1-Year	92	346	77	273	15	73	0.38	0.25
2-Year	90	340	74	270	16	70	0.66	0.18
3-Year	90	336	65	275	25	61	0.06	1.16
4-Year	88	329	72	261	16	68	0.71	0.18
5-Year	83	219	70	251	13	68	0.32	0.30
10-Year	78	295	64	234	14	61	0.71	0.19
**White or other race**								
1-Year	346	92	273	77	73	15	0.38	0.25
2-Year	340	90	270	74	70	16	0.66	0.18
3-Year	336	90	275	65	61	25	0.06	1.16
4-Year	329	88	261	72	68	16	0.71	0.18
5-Year	319	83	251	70	68	13	0.32	0.30
10-Year	295	78	234	64	61	14	0.71	0.19
**Outcome Variable**								
**Survival duration**								
1-Year	405	33	324	26	81	7	> 0.99	0.10
2-Year	330	100	264	80	66	20	> 0.99	0.16
3-Year	269	157	215	125	54	32	> 0.99	0.19
4-Year	223	194	178	155	45	39	> 0.99	0.19
5-Year	181	221	145	176	36	45	> 0.99	0.20
10-Year	71	302	57	241	14	61	> 0.99	0.16

^*^P values determined using Pearson’s chi-squared test

### Model development

We selected gradient boosting machine (GBM) modeling because it is a decision tree machine learning technique that builds an ensemble of shallow and weak trees or learners in succession (rather at than all at once as in random forest machine learning), so each tree learns and improves from the previous iteration. GBM modeling trains models in a gradual, additive, and sequential manner, which strengthens the final product [18, 19]. The final model is built on the strength of previous, smaller predictors.

We used Python, version 3.7.4 (Python Software Foundation, Beaverton, OR) to develop the models. For each model, we split the data 80/20 into train and test groups and a further 80/20 split of the train group into train and validation sets stratified by the binary outcome feature across all groups. Data were shuffled to create random order before splitting into listed groups. Because the number of patients at each time point differed, the exact number of records in the train, validation, and test groups vary by time point and cohort. Data types were changed to integer, float, and object as applicable. Missing data were imputed using the multiple imputation by chained equations algorithm from the IterativeImputer package. Our data were preprocessed to scale using the MinMaxScaler package of sklearn.preprocessing. Six GBM models were created, 1 for each of the 6 survival time points, using the train set and the GradientBoostingClassifier package in sklearn.ensemble.

### Feature selection

Because of our limited data set, we made all categorical features binary. This allowed for a more transparent analysis of results. We performed feature selection using Boruta Random Forest algorithm; all features were included. Features were further characterized for magnitude and direction of each feature’s association with the outcome (patient survival) using the local interpretable model–agnostic explanations (LIME) package in R software for the models [20].

### Model regulation

GBM models continue improving to minimize error at the risk of overfitting. For internal validation, we used a cross-validated grid search to direct our choice of parameters using GridSearch CV within sklearn.model_selection package (Python Software Foundation). Our scoring measure of interest was the AUC. For each model, we selected parameters that produced the highest AUC in the validation set. Parameters of interest were learning rate, number of estimators, maximum depth of tree, minimum number of samples per node to be considered for splitting, minimum number of samples required in a terminal node, and subsample percentage included for each tree.

### Performance assessment

We created predictive values for each model by using each corresponding test set. First, calibration plots were used to visualize the concurrence of the predicted probabilities with the observed frequencies in the data set. Then, discriminatory ability was determined by estimating the AUC. Next, Brier scores were used to determine overall accuracy of the predictions. The Brier score measures distance between the actual outcome and the predicted probability assigned to the outcome for each observation, where the best possible Brier score for accuracy is 0 and the worst is 1 [21, 22]. Finally, we determined whether the models possessed clinical utility by using decision curve analysis [23, 24], as described previously in this patient population [12].

## Results

### Participants

Continuous and categorical features for the train and test sets are listed in Tables 1 and 2, respectively. As expected, we found no difference between continuous features in the train and test sets (BF of approximately 0) (Table 1). When comparing categorical features, we found no difference between the 2 groups (BF of approximately 0) for treatment type and survival (yes/no) at any time point (Table 2).

### Model development and validation

**Fig. 1:**
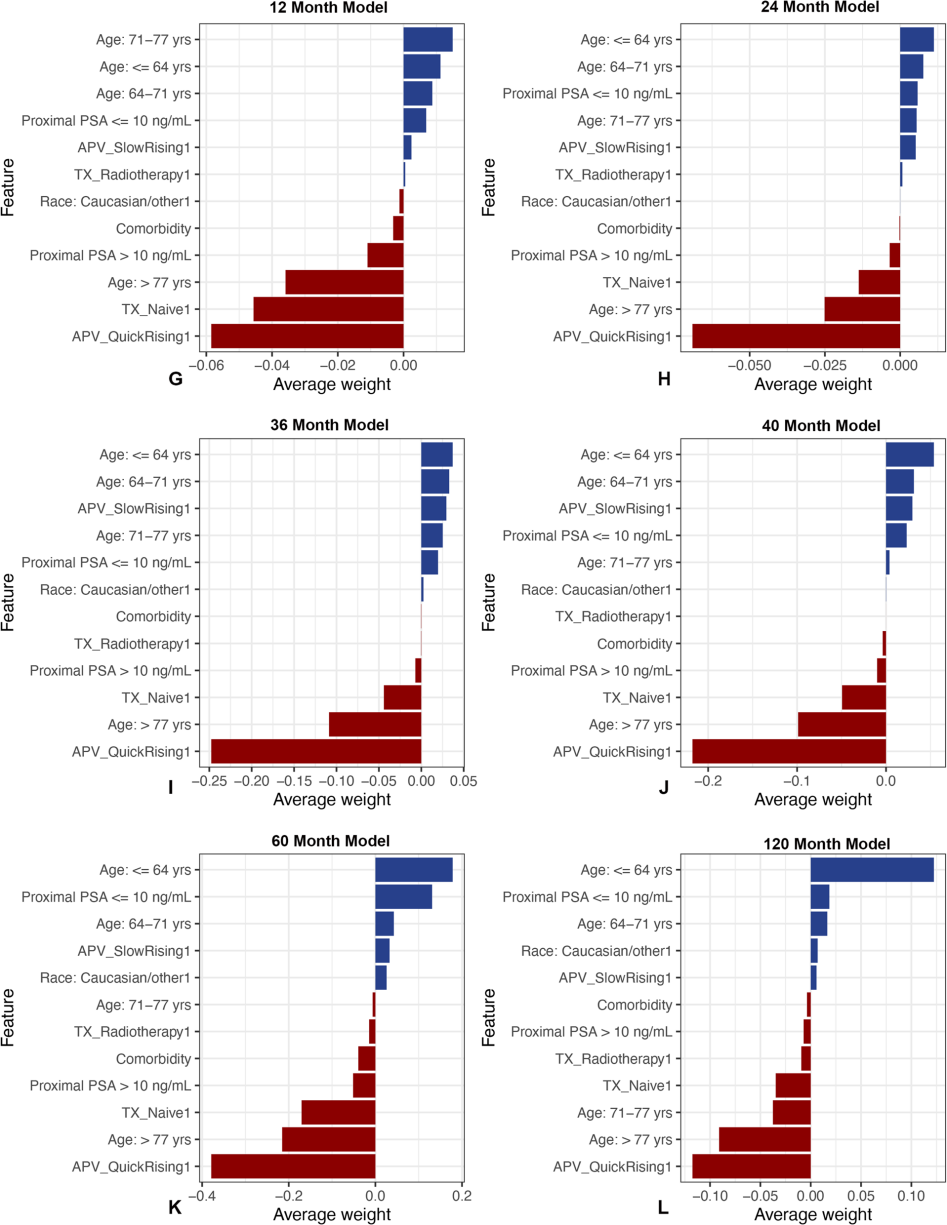
**A-F** This figure shows both the relative influence of each feature and whether the feature has a positive or negative association with survival. The directionality (to support or contradict the outcome of interest) of each level of the model features is ranked by average weight of feature level across all cases. Blue bars (positive feature weight) are associated with features that are associated with survival; red bars (negative feature weight) represent features that are negatively associated with survival at (**A**) 1 year, (**B**) 2 years, (**C**) 3 years, (**D**) 4 years, (**E**) 5 years, and (**F**) 10 years

The AUC was between 0.73 and 0.86 for all 6 models (Table 3). Brier scores were < 0.20 and demonstrated the model’s predictions were accurate. The relative influence table for the 6 models in Fig. 1 shows the degree of influence for each feature on the overall model. Proximal PSA and patient age at the time of first-known SRE consistently had the most influence across all models. Treatment method and APV became increasingly influential with the later time period models.

**Table 3:** Summary of the accuracy (AUC) and discriminatory ability (Brier score) of the predictive model at each time period

Model	AUC (95% CI)	Brier Score (95% CI)
1-Year	0.76 (0.61–0.91)	0.07 (0.02–0.12)
2-Year	0.73 (0.60–0.85)	0.17 (0.12–0.22)
3-Year	0.86 (0.79–0.94)	0.19 (0.16–0.21)
4-Year	0.82 (0.73–0.91)	0.20 (0.18–0.22)
5-Year	0.79 (0.69–0.89)	0.19 (0.15–0.23)
10-Year	0.79 (0.65–0.93)	0.14 (0.09–0.19)

*AUC* area under the receiver operating characteristic curve, *CI* confidence interval

### Global application (Model-level interpretation)

In earlier survival estimate models, proximal PSA and age at diagnosis had more influence on the outcome variable. Notably, APV was an important feature at all time points; quick-rising APV was more influential in later survival estimate mode. Unexpectedly, the method of treating the primary disease also had strong influence; however, treatment-naïve status was more influential on survival than was radiotherapy and/or chemotherapy.

### Local application (Patient-level interpretation)

To trust and apply models correctly, clinicians must be able to interpret them at the patient level [20]. The positive and negative directionality of each model is shown in Fig. 1. Overall, features positively associated with survival were younger age at metastasis diagnosis, proximal PSA < 10 ng/mL, slow-rising APV, no-rising APV, radiotherapy treatment, and hormonal or chemotherapy treatment (Fig. 1). Features negatively associated with survival were older age at metastasis diagnosis, proximal PSA > 10 ng/mL, quick-rising APV values, and being treatment-naïve (Fig. 1). The patient-level interpretations were consistent with global-level model application.

### Clinical utility

**Fig. 2:**
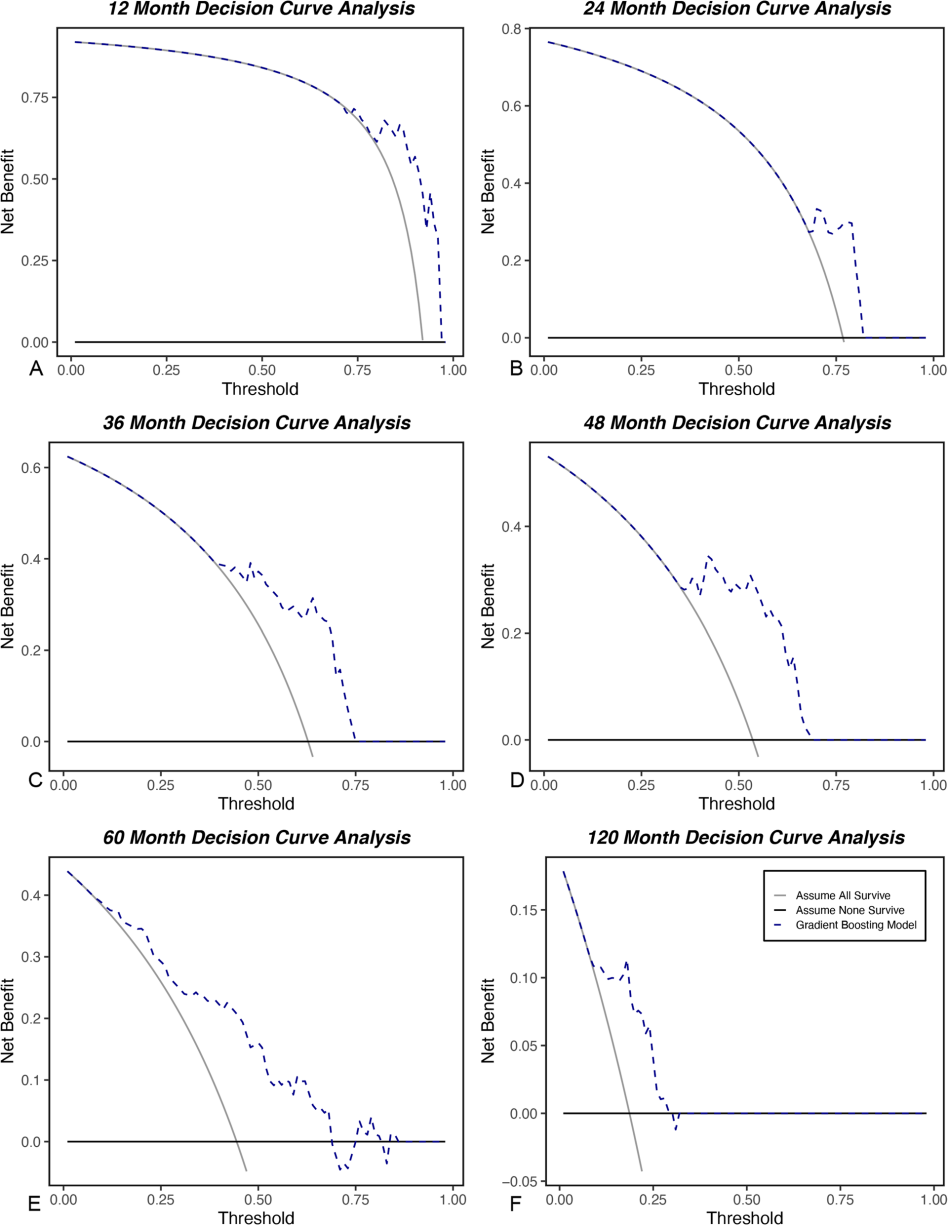
**A-F** Decision curve analyses of each of the 6 models designed to estimate patient survival at (**A**) 1 year, (**B**) 2 years, (**C**) 3 years, (**D**) 4 years, (**E**) 5 years, and (**F**) 6 years after treatment or surgery for skeletal-related events due to bone metastasis from prostate cancer. These results suggest that all the models (dotted line) should be used rather than assuming all patients (continuous line) or no patients (thick continuous line) will survive longer than the period of each predictive model

Decision curve analysis showed that physicians may achieve better outcomes by using the 6 models described above, rather than assuming all will survive, or none will survive for 1, 2, 3, 4, 5, and 10 years, respectively (Fig. 2). Decision curve analysis measures the net benefit of using a clinical support tool across different threshold probabilities defined as the point of equipoise when considering 2 courses of treatment (e.g., nonsurgical vs. surgical for short-term survival estimates, less invasive vs. more invasive for longer-term estimates). Low-threshold probabilities are associated with healthier patients, whereby physicians have a low threshold to offer surgery. In contrast, high-threshold probabilities are associated with patients in which surgery carries greater risk.

## Discussion

The duration of survival for prostate cancer patients with metastatic bone disease is difficult to predict. We successfully developed models to estimate survival in patients with prostate cancer who have metastatic bone disease to help clinicians navigate treatment algorithms. Previous studies have shown that APV is predictive of distant metastasis–free survival [4, 5, 25]. In this study, we showed that machine learning–based models can predict survival in prostate cancer patients, and that these models improve in both discriminatory ability and accuracy with the addition of APV data. Specifically, the patient’s primary disease treatment type and APV became increasingly influential in the later time period models. Although further external validation studies are required, these data justify inclusion of these models in the PATHFx tool, an open-source clinical decision-making support tool for survival estimation (https://www.pathfx.org) [7].

Patient race and ethnicity may provide important information on genetic and socioeconomic factors pertaining to disease [26, 27]. Race was self-reported by patients at the time of enrollment and divided into 2 broad categories (white/other or black). Using the CPDR database, Cullen et al. [28] found self-reported black race was not a predictor of poorer overall survival among participants in the CPDR Multicenter National Database Program undergoing active surveillance, despite race-based differences in baseline clinical risk characteristics.

Although PATHFx is validated, it does not offer disease-specific estimates of survival [12]. The prostate cancer–specific models at 1 and 2 years can be compared directly with the PATHFx 1- and 2-year models in terms of discriminatory ability (AUC) and accuracy of prediction (Brier score). The new 1-year prostate disease–specific model we developed (AUC = 0.85; Brier score = 0.07) was superior to the PATHFx (version 3.0) 1-year model (AUC = 0.78; Brier score = 0.18). However, the 2-year prostate disease–specific model (AUC = 0.80; Brier score = 0.17) was no better than the PATHFx 2-year model (AUC = 0.82; Brier score = 0.12). Based on this direct comparison, the 1-year prostate disease–specific model could be used independently to accurately determine survival duration in men with metastatic prostate cancer. However, predictive algorithms continue to improve with exposure to more data [29]. Therefore, we believe there is room for improvement by incorporating additional PATHFx variables, such as hemoglobin concentration and absolute lymphocyte count.

Although the classification ability of the prostate-specific models derived in this study is no better than that of the current PATHFx tool [12], we have developed 4 additional models that estimate survival at 3, 4, 5, and 10 years. Validation statistics and decision curve analysis indicate that these models are suitable for clinical use. Incorporating these prostate cancer–specific models into the PATHFx clinical support tool is part of our continued responsibility to provide accurate estimations of survival to help clinicians and patients navigate complex treatment algorithms. Unlike traditional statistical decision rules, the accuracy of machine learning–based models can be improved over time with better machine learning methods, more data, changes in practice, changes in the patient population, and/or better understanding of disease processes [29].

When evaluating the results of this study, its limitations must be considered. It is possible that other statistical techniques could be used to develop similar prognostic models for prostate cancer. Our author group has extensive experience using various machine learning techniques. Some techniques are prone to overfitting and produce overly optimistic results. Therefore, we implemented GBM with hyperparameter tuning to mitigate the risk of overfitting. Our study was limited by missing data, which can result in incomplete codification of train data and overfitting; however, we mitigated these effects by using a “holdout” validation set. Despite these results, external validation studies are necessary before these models can be recommended for use in other patient populations. Beyond APV, there may be other laboratory-related features to consider for use in the model; however, the data are not complete in the CPDR database. The number of features available for the model was a limitation. Only 31% of the CPDR data had APV data, so we restricted the data set to the 438 records with APV values. Nevertheless, we expect the models to continue to improve as more data become available.

Although the addition of APV to the models improved performance, we may see continued improvement in model performance by including additional demographic and laboratory-based patient data. For example, Stattin et al. [30] found that a panel of kallikrein marker (human kallikrein-related peptidase 2 [hK2] and total, free, and intact PSA) is strongly predictive of distant metastasis in men with modestly elevated PSA. As these data are collected and added to national and international prostate cancer registries, we could continue to augment the models for survival estimations. Additionally, different mechanisms exist to measure and categorize APV, and previously determined [4, 5, 25] cut points were used in this analysis. Furthermore, PSMA PET and bone scintigraphy have been shown to predict the survival of end-stage prostate cancer patients [31]. It possible that integrating APV into PATHFx with these imaging biomarkers may further strengthen survival estimates.

By including disease-specific information such as APV, we have developed a tool that helps predict survival duration in men with metastatic bone disease due to prostate cancer. Although external validation studies are required to support its use in other patient populations, these data justify inclusion of these models in the PATHFx tool. In addition, data used in the GBM model, including APV and proximal PSA, should be included in the International Bone Metastasis Registry. 

## Data Availability

The datasets generated and/or analyzed during the current study are not publicly available due to Department of Defense regulations, but may be available from the corresponding author on reasonable request.

## Abbreviations

APV: Alkaline Phosphatase Velocity
AUC: Area Under The Curve
BF: Bayes Factor
CPDR: Center for Prostate Disease Research
GBM: Gradient Boosting Machine
LIME: Local Interpretable Model–Agnostic Explanations
PSA: Prostate-Specific Antigen
SRE: Skeletal-Related Event
